# Preliminary functional outcomes after staged heterotopic groin-pocket salvage following intraoperative thumb replantation failure: a four-patient technical case series

**DOI:** 10.3389/fsurg.2026.1879687

**Published:** 2026-07-30

**Authors:** Ovunc Akdemir, Atilla Adnan Eyuboglu, Feng Zhang, William C. Lineaweaver

**Affiliations:** 1 Department of Plastic, Reconstructive and Aesthetic Surgery, Istanbul Aydin University, Istanbul, Türkiye; 2 Department of Plastic, Reconstructive and Aesthetic Surgery, Istanbul Arel University, Istanbul, Türkiye; 3 Department of Plastic and Reconstructive Surgery, Vanderbilt University Medical Center, Nashville, TN, United States

**Keywords:** functional outcomes, groin-pocket salvage, heterotopic banking, microsurgery, pinch strength, salvage reconstruction, secondary reconstruction, thumb replantation

## Abstract

**Background:**

Intraoperative circulatory failure during thumb replantation represents a rare and challenging reconstructive scenario for which established salvage strategies and objective functional outcome data remain limited. We describe the technical feasibility and preliminary 12-month functional outcomes of a staged heterotopic groin-pocket salvage strategy in a four-patient retrospective clinical series.

**Methods:**

This retrospective technical feasibility case series included four highly selected patients with traumatic thumb amputations at the interphalangeal joint level who underwent staged heterotopic groin-pocket salvage after intraoperative replantation failure. The procedure incorporated preservation of the native flexor pollicis longus and extensor pollicis longus tendon insertion sites, delayed skeletal stabilization, staged tendon reconstruction, and adjunctive placement of a carboxymethylcellulose–hyaluronan adhesion barrier. Outcomes assessed at 12 months included interphalangeal and metacarpophalangeal joint motion, tip-to-tip pinch strength, QuickDASH score, static two-point discrimination, aesthetic satisfaction, and radiographic joint appearance. Published thumb-reconstruction outcomes were reviewed solely to provide descriptive clinical context.

**Results:**

At 12 months, mean interphalangeal flexion was 71.3°, mean metacarpophalangeal flexion was 47.5°, and mean tip-to-tip pinch strength was 4.38 kg, corresponding to 96.3% of the contralateral side. The mean QuickDASH score was 3.75, and mean static two-point discrimination was 9.5 mm. Mean aesthetic satisfaction was 2.0 on a 5-point scale, in which lower scores indicated greater satisfaction. Mild joint-space narrowing was observed radiographically at the available follow-up. No inferential statistical comparisons with published series were performed because of the small cohort size and substantial clinical and methodological heterogeneity across the literature.

**Conclusions:**

In this small, anatomically homogeneous, and highly selected series, staged heterotopic groin-pocket salvage was technically feasible and was associated with preserved thumb length and functional use at 12 months. These preliminary observations do not establish comparative effectiveness, procedural superiority, reproducibility, or long-term durability. Larger clinical series and longer follow-up are required before the technique can be considered a reproducibly effective reconstructive strategy.

## Introduction

Thumb replantation remains the preferred reconstructive strategy following traumatic thumb amputation because of the thumb's essential role in pinch, opposition, and coordinated hand function. Advances in microsurgical technique and perioperative management have substantially improved digital replantation survival over recent decades, with successful revascularization reported in more than 80% of carefully selected cases (1). Even partial preservation of thumb length and mobility may significantly improve upper-extremity performance, dexterity, and overall quality of life (2, 3). Consequently, restoration of a functional thumb remains a central objective in the management of severe hand trauma.

Despite these advances, intraoperative circulatory collapse during thumb replantation continues to represent one of the most challenging salvage scenarios in reconstructive microsurgery. In high-energy crush or avulsion injuries, extensive endothelial disruption, segmental vascular destruction, and progressive microvascular thrombosis may render repeated arterial or venous revision unsuccessful despite technically adequate anastomoses. Under these circumstances, surgeons are frequently confronted with the difficult decision between prolonged but futile revascularization attempts and immediate revision amputation, both of which may ultimately compromise long-term functional recovery.

Temporary heterotopic implantation has been described as a salvage strategy for complex traumatic amputations in situations where immediate definitive reconstruction is biologically or technically unfavorable (4, 5). Godina et al. (6) originally introduced ectopic implantation as a temporizing approach for severely mutilated extremity injuries in which preservation of viable tissue was prioritized over immediate anatomic reconstruction. By transferring the amputated segment into a well-vascularized ectopic recipient site, tissue viability may be maintained while definitive skeletal, tendon, and soft-tissue reconstruction is delayed until more favorable biological conditions are established. Subsequent reports have supported the use of heterotopic implantation in selected thumb injuries, particularly in the setting of severe soft-tissue destruction, unstable recipient vessels, or progressive vascular insufficiency (7).

However, the available literature remains limited regarding functional outcomes after heterotopic salvage performed specifically for intraoperative thumb replantation failure. Most published reports primarily emphasize technical feasibility or survival of the amputated segment, whereas objective postoperative outcomes—including interphalangeal motion, pinch strength, disability scores, and patient-reported recovery—remain insufficiently characterized. Furthermore, comparative functional data contextualizing heterotopic salvage relative to contemporary thumb reconstruction outcomes remain sparse in the current literature.

The purpose of this study was therefore to present a staged heterotopic groin-pocket salvage strategy following intraoperative thumb replantation failure and to evaluate its functional outcomes in a four-patient clinical series in the context of currently reported thumb reconstruction outcomes. Objective postoperative evaluation included interphalangeal and metacarpophalangeal motion, tip-to-tip pinch strength, sensibility, QuickDASH scores, and patient-reported aesthetic satisfaction at 12 months after reconstruction.

## Materials and methods

### Ethical considerations

This retrospective study was reviewed by the Istanbul Aydın University Institutional Review Board, which determined that formal ethical approval was not required in accordance with institutional policy. Written informed consent for the use of clinical data was obtained from all participants. Separate written informed consent for the publication of identifiable clinical photographs was also obtained from each participant. The study was conducted in accordance with the Declaration of Helsinki.

### Patient selection

Between 2012 and 2024, a total of 378 digital replantation procedures were performed at our institution. Of these, 52 cases (13.8%) involved thumb replantation, highlighting the critical functional importance of the thumb in hand biomechanics. Among the 52 cases, 4 patients (7.7%)—three males and one female, aged 28–49 years—sustained traumatic thumb amputations and subsequently experienced intraoperative replantation failure due to irreversible endothelial damage and circulatory insufficiency.

Three patients were injured in high-energy industrial crush accidents, while one sustained a traumatic amputation in a motor vehicle collision. In all cases, initial microsurgical replantation attempts were performed; however, despite technically successful anastomoses, critical vascular failure ensued. Indications for heterotopic salvage included persistent intraoperative vascular insufficiency despite repeated microsurgical revision, preservation of viable tendon and soft-tissue architecture, and absence of irreversible contamination or proximal tissue destruction. Rather than proceeding with primary amputation or stump closure, a decision was made to attempt salvage via heterotopic implantation in the groin region.

For each patient, the heterotopic pocket was created in the ipsilateral groin, corresponding to the side of the injured thumb. In all four patients, the level of thumb amputation was located at the interphalangeal (IP) joint level.

### Surgical technique

In all four patients, despite multiple intraoperative attempts at arterial and venous anastomosis, adequate circulation could not be achieved. Following failed microsurgical replantation, the amputated thumb segment was immediately prepared for heterotopic implantation. The soft tissues surrounding the distal phalanx were sharply debrided using a No. 15 scalpel and scissors, while preserving the viable distal phalanx and the native insertion sites of the flexor pollicis longus (FPL) and extensor pollicis longus (EPL) tendons. The resulting osteotendinous segment was then prepared for implantation into the ipsilateral groin pocket. The volar and dorsal skin dimensions of the contralateral healthy thumb were measured with a sterile paper ruler. These dimensions were transposed to the ipsilateral groin region, where a skin island matching the thumb's width and length was outlined. A subcutaneous pocket was then created beneath the marked skin using blunt dissection and scissors, with the entry centered at the proximal base of the outline.

The debrided amputated tissue was inserted into the pocket and secured using 3/0 polypropylene (Prolene) sutures. The original FPL and EPL tendons at the donor site were tagged with 1/0 polypropylene and temporarily sutured to the surrounding soft tissue to prevent retraction or malalignment. The skin defect on the original thumb stump was closed primarily with 3/0 polypropylene sutures.

On postoperative day 10, the stump incision was reopened and the groin pocket was accessed. A carboxymethylcellulose–hyaluronan adhesion barrier (SEPRAFILM®) was used off-label. The sheet was trimmed to fit the exposed peri-IP region and placed around the periarticular tissue and bone surfaces before temporary transarticular Kirschner-wire stabilization, with the aim of limiting periarticular adhesion formation. The barrier was left *in situ* for resorption. All four patients received the material. Under ultrasound guidance, a Kirschner wire was advanced through the preserved distal phalanx segment of the amputated thumb and into the proximal phalanx stump to achieve temporary skeletal stabilization and alignment. Correct intramedullary positioning of the Kirschner wire was confirmed using both ultrasound visualization and direct tactile palpation. The skin of the groin flap was aligned with the hand stump and fixed using 3/0 polypropylene sutures. To minimize tension on the flap, the hand was secured to the groin with a 1/0 silk suture extending from the wrist to the groin. These fixation sutures were removed on postoperative day 20.

On postoperative day 21, the composite osteocutaneous unit consisting of the groin flap and preserved distal phalanx segment was elevated *en bloc* following division of the volar pedicle. The flexor pollicis longus tendon was identified along the volar aspect of the preserved distal phalanx and repaired to its corresponding proximal stump using a modified Kessler technique with 3-0 polypropylene sutures. Formal collateral ligament or volar plate reconstruction was not performed. Instead, the residual periarticular soft tissues together with the surrounding subcutaneous groin-flap tissues were strategically approximated around the reconstructed interphalangeal region using absorbable sutures to provide biologic soft-tissue reinforcement and secondary stabilization during healing. The skin was subsequently closed using 3-0 polypropylene sutures.

**Figure 1:**
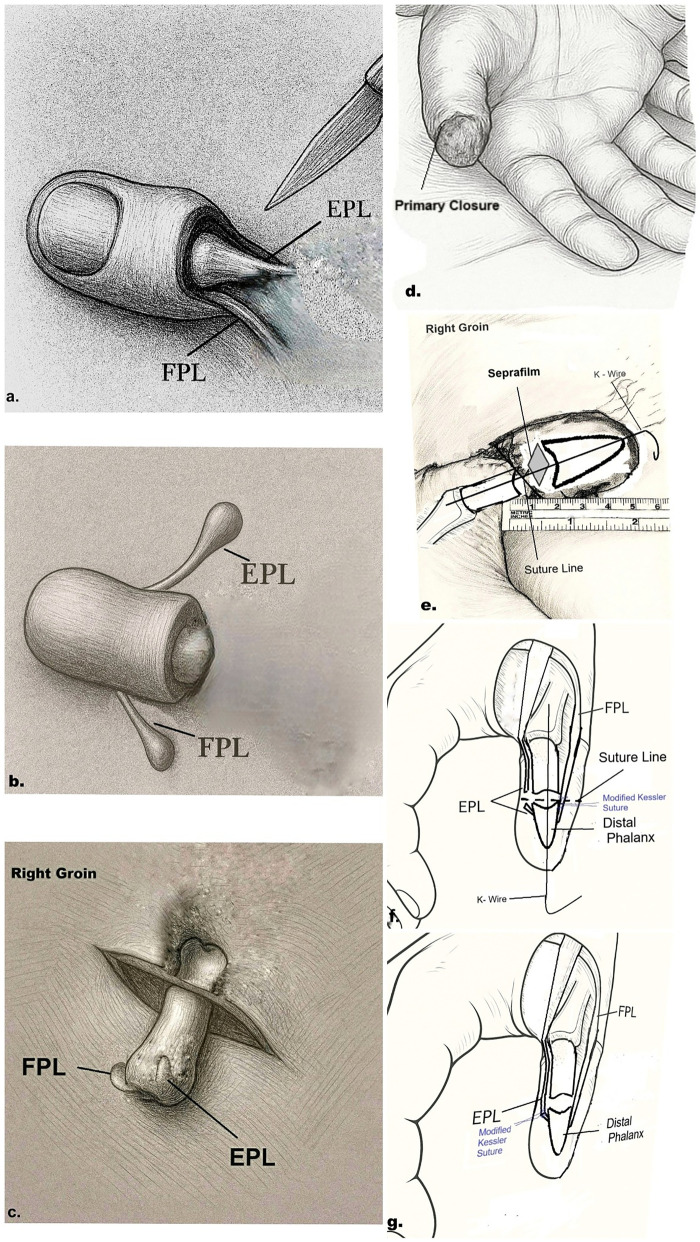
Schematic illustration of the staged heterotopic groin-pocket salvage technique following intraoperative thumb replantation failure. **(a)** Debridement of the amputated thumb at the interphalangeal (IP) level while preserving the flexor pollicis longus (FPL) and extensor pollicis longus (EPL) tendon insertion sites. **(b)** Preparation of the distal phalanx segment with preservation of the native FPL/EPL attachment zones. **(c)** Heterotopic implantation of the composite distal phalanx segment into an ipsilateral groin pocket prepared slightly wider than the osseous dimensions. **(d)** Primary closure of the proximal thumb stump after implantation. **(e)** On postoperative day 10, the groin pocket is reopened, Carboxymethylcellulose Hyaluronan (SEPRAFILM®) is applied off-label around the peri-IP region with the aim of limiting periarticular adhesion formation, and temporary skeletal stabilization is achieved using Kirschner-wire fixation under ultrasound guidance. **(f)** On postoperative day 21, the osteocutaneous construct is elevated *en bloc* following division of the volar pedicle, and the FPL tendon is repaired using a modified Kessler technique. **(g)** On postoperative day 28, the Kirschner wire is removed, followed by delayed EPL repair and dorsal skin closure.

**Figure 2:**
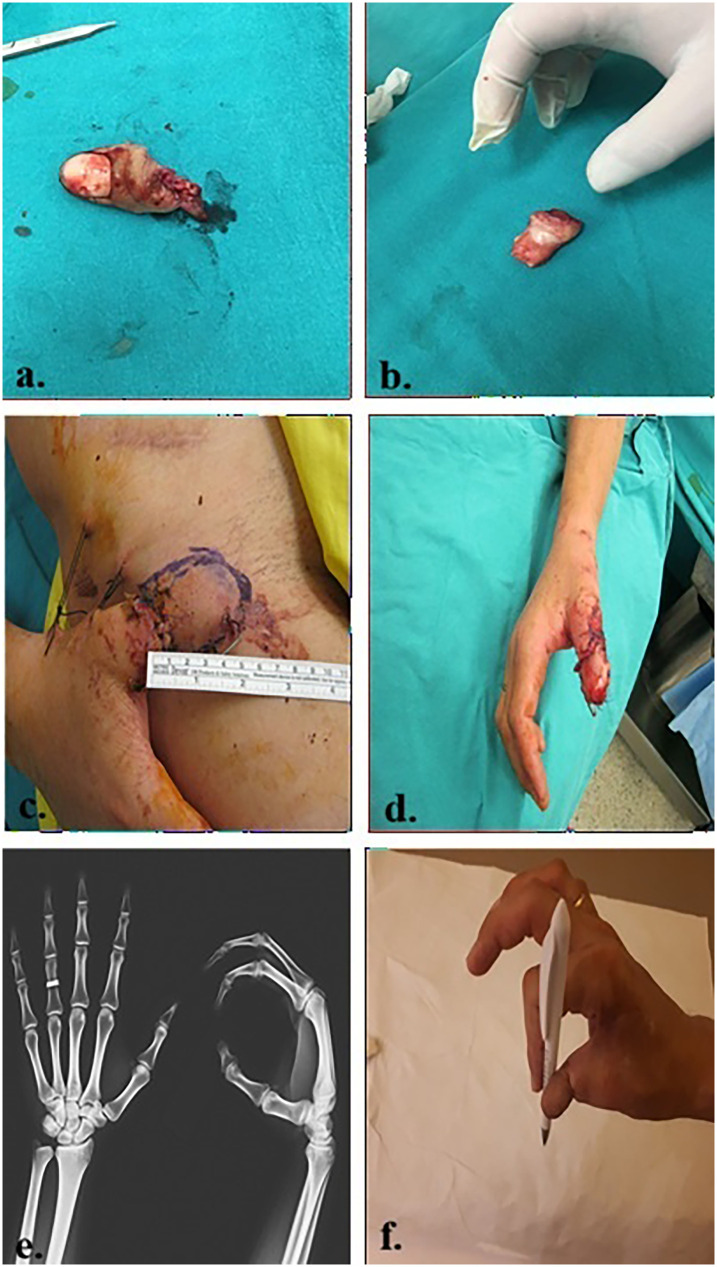
Clinical sequence demonstrating staged heterotopic groin-pocket salvage following intraoperative thumb replantation failure in a 42-year-old male patient with traumatic right-thumb amputation. **(a)** Amputated thumb following failed replantation attempt. **(b)** Debridement of the distal phalanx segment with preservation of the flexor pollicis longus (FPL) and extensor pollicis longus (EPL) tendon insertion sites. **(c)** Reopening of the groin pocket on postoperative day 10 for SEPRAFILM® application around the peri-interphalangeal region and temporary skeletal stabilization using Kirschner-wire fixation under ultrasound guidance. **(d)** Clinical appearance during staged reconstruction prior to pedicle division. **(e)** One-year postoperative radiograph showing mild joint-space narrowing without gross articular collapse or advanced degenerative change. **(f)** Demonstration of functional precision pinch at 12 months, showing the patient holding a pen with mild compensatory hyperextension.

On postoperative day 28, the Kirschner wire was extracted. Under local anesthesia, the dorsal ends of the extensor pollicis longus tendons were identified and repaired using a modified Kessler technique with 3/0 polypropylene. The dorsal skin was closed with 4/0 polypropylene sutures (Figures 1, 2). Additional intraoperative and postoperative clinical views are provided in Supplementary Figure S1.

### Postoperative rehabilitation

No additional external splint was used, as temporary skeletal and joint stabilization during the initial postoperative period was provided by the transarticular Kirschner wire. Following Kirschner-wire removal and completion of EPL repair on postoperative day 28, gentle passive MCP and IP range-of-motion exercises were initiated after one week. Controlled active thumb-motion exercises were introduced at three weeks. Light, non-resisted tip-to-tip pinch coordination exercises were initiated at the end of the third week. No formal sensory re-education protocol was implemented.

### Postoperative evaluation

Patients underwent standardized postoperative rehabilitation and were followed for a minimum of 12 months. All objective outcome assessments, including range of motion, tip-to-tip pinch strength, joint stability, and static two-point discrimination, were performed independently by the operating senior microsurgeon and a second hand surgeon who had not participated in the patients' treatment. The second examiner was blinded to the operating surgeon's measurements and to the patients' previously recorded functional outcome data. Any discrepancies between the two assessments were resolved by consensus. Functional assessments at 12 months included:

- Range of Motion (ROM):

Measured using a standard goniometer for the metacarpophalangeal (MCP) and interphalangeal (IP) joints. Flexion and extension angles were recorded.

- Pinch Strength:

Tip-to-tip pinch strength was measured using a calibrated Saehan SH5005 pinch gauge. Patients were seated with the shoulder in a neutral position, the elbow flexed at 90°, and the forearm in neutral rotation. Each examiner independently obtained three consecutive maximal-effort trials, and the mean of the two examiners' averaged measurements was used for analysis.

- Patient-Reported Outcomes:

The Quick-Disabilities of the Arm, Shoulder and Hand (QuickDASH) questionnaire was administered. In addition, patients were asked to rate their aesthetic satisfaction with the reconstructed thumb on a 5-point Likert scale (1 = very satisfied, 5 = very dissatisfied).

- Joint Stability and Pain Assessment:

At the 12-month follow-up, the reconstructed IP joint was assessed clinically for lateral stability using manual radial–ulnar stress, rotational stability, and pain during maximal-effort tip-to-tip pinch. Findings were recorded as present or absent, as no instrumented stress-testing device or validated pain scale was used.

- Sensory Evaluation:

Sensory assessment was performed at the 12-month follow-up. Static two-point discrimination (s2PD) was measured at the pulp of the reconstructed thumb using a Dellon discriminator, following standard hand-surgery protocols. With the patient's eyes occluded, the smallest distance at which two points were perceived as separate in ≥50% of trials was recorded as the s2PD value.

- Radiographic Evaluation:

Available 12-month radiographs were independently reviewed by two radiologists who were blinded to the patients' clinical and functional outcomes and to each other's assessments. Radiographs were evaluated for joint-space narrowing, articular collapse, osteonecrosis or resorption of the preserved phalangeal segment, and other degenerative changes. Any discrepancies between the two assessments were resolved by consensus.

### Contextual literature comparison

To provide descriptive clinical context for the postoperative outcomes observed in the present series, a structured literature search was performed using PubMed, Scopus, and Web of Science. Search terms included combinations of “thumb amputation”, “thumb replantation failure”, “heterotopic implantation”, “ectopic thumb transfer”, “secondary thumb reconstruction”, “groin-pocket salvage”, “thumb range of motion”, “pinch strength”, and “QuickDASH”.

Studies published between 1990 and 2024 were screened for quantitative functional outcomes following traumatic thumb reconstruction, replantation, heterotopic salvage, or secondary reconstructive procedures. Studies were considered relevant when they reported one or more of the functional domains evaluated in the present series, including thumb interphalangeal motion, tip-to-tip pinch strength, and QuickDASH scores.

Because the identified studies differed substantially in injury mechanism, amputation level, reconstructive technique, patient selection, follow-up duration, rehabilitation protocol, and outcome-measurement methodology, published values were used solely as descriptive contextual references. No pooled literature estimate or formal statistical comparison was performed. The published series were not treated as a control group and were not used to establish comparative effectiveness, equivalence, or superiority.

### Descriptive analysis

Owing to the small cohort size (*n* = 4), the analysis was limited to descriptive reporting. Objective and patient-reported outcomes were summarized using mean and range, as appropriate. No inferential statistical testing, hypothesis testing, confidence-interval estimation, or sample-size calculation was performed.

## Results

### Cohort characteristics

Four patients (three male and one female; mean age, 39.5 years; range, 28–49 years) underwent staged heterotopic groin-pocket salvage after intraoperative thumb replantation failure. Injury mechanisms included three high-energy industrial crush injuries and one motor vehicle collision. In all four patients, the amputation level was at the interphalangeal (IP) joint level.

### Objective and patient-reported outcomes at 12 months

Objective and patient-reported outcomes at the 12-month follow-up are summarized descriptively in Table 1.

**Table 1:** Descriptive objective and patient-reported outcomes at 12 months in the present cohort (*n* = 4).

Outcome	Value
MCP flexion (°)	47.5 (45–50)
IP flexion (°)	71.25 (55–80)
MCP extension (°)	0° (full extension) in all patients
IP extension/hyperextension (°)	0° in two patients; +10° hyperextension in two patients
Combined MCP and IP flexion (°)	118.75
Tip-to-tip pinch strength (kg)	4.375 (3.5–5.0)
Pinch strength relative to the contralateral side (%)	96.25 (90–100)
QuickDASH score (0–100; lower scores indicate less disability)	3.75 (1–7)
Static two-point discrimination (mm)	9.5 (7–12)
Aesthetic satisfaction (1 = very satisfied; 5 = very dissatisfied)	2.0 (1–3)

MCP, metacarpophalangeal; IP, interphalangeal; QuickDASH, quick disabilities of the arm, shoulder and hand.

Values are presented as mean and range unless otherwise indicated. Positive IP extension values indicate hyperextension. Because of the small cohort size, no inferential statistical testing was performed.

Mean metacarpophalangeal (MCP) flexion was 47.5° (range, 45°–50°), and mean IP flexion was 71.25° (range, 55°–80°). Full MCP extension was present in all patients. Two patients demonstrated neutral IP extension, whereas two demonstrated 10° of IP hyperextension. Mean combined MCP and IP flexion was 118.75°.

**Figure 3:**
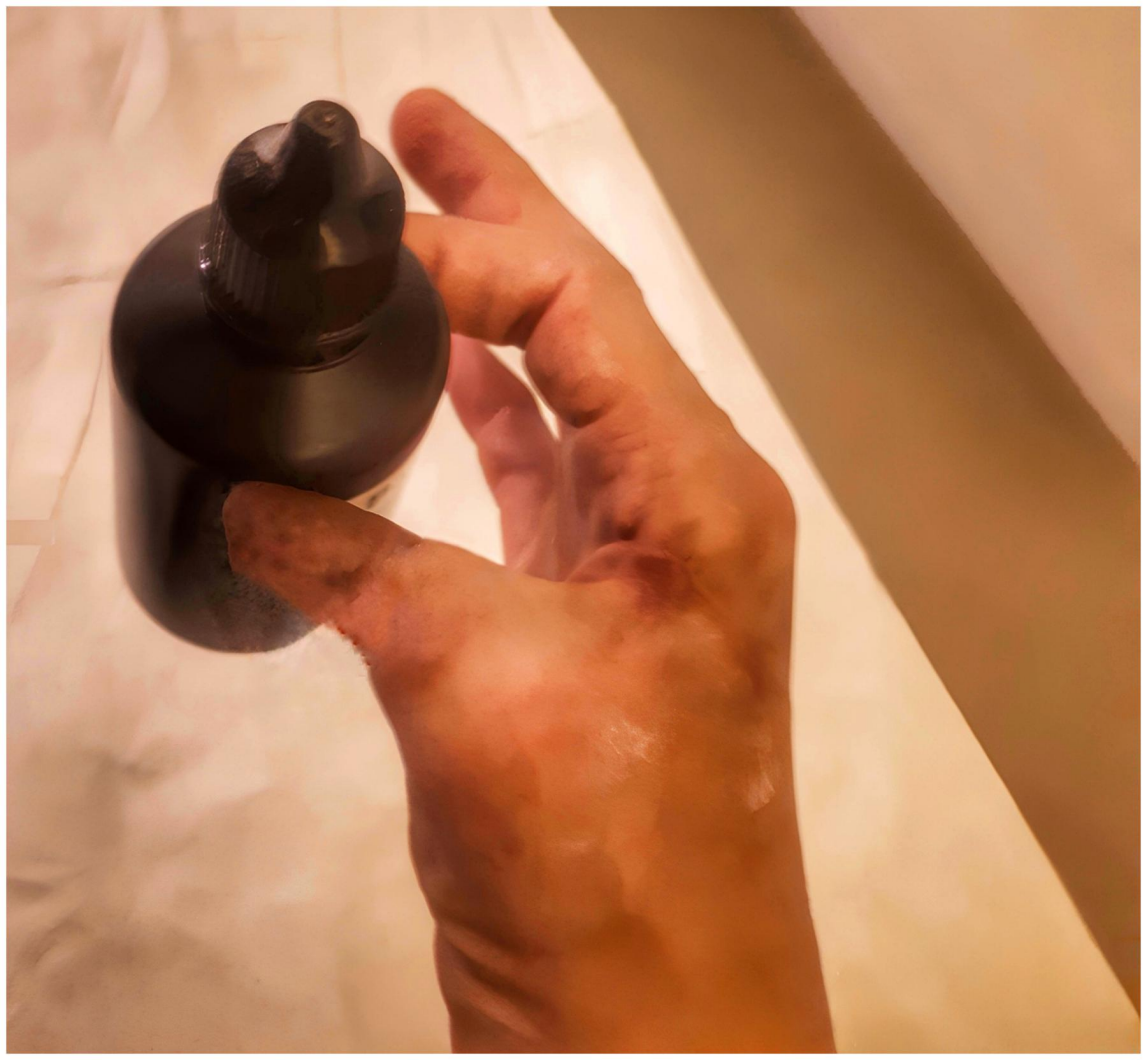
Functional demonstration of tip-to-tip pinch ability at 12 months in a 39-year-old male patient who sustained a high-energy industrial crush injury. Although quantitative pinch measurements were obtained using a calibrated pinch gauge, this illustrative functional demonstration shows the patient lifting a 100-cc povidone–iodine bottle, reflecting practical postoperative pinch performance.

Mean tip-to-tip pinch strength was 4.375 kg (range, 3.5–5.0 kg), corresponding to 96.25% (range, 90%–100%) of the contralateral side. In addition to measurements obtained using a calibrated pinch gauge, functional pinch ability was illustrated by lifting a 100-cc povidone–iodine bottle (Figure 3). This demonstration was not included among the quantitative outcome measurements. At the 12-month examination, no clinically evident lateral or rotational instability of the reconstructed IP joint was observed in any patient. None of the patients reported pain during maximal-effort tip-to-tip pinch testing.

The mean QuickDASH score was 3.75 (range, 1–7). Mean aesthetic satisfaction was 2.0 (range, 1–3) on a 5-point Likert scale, in which lower scores indicated greater satisfaction. Mean static two-point discrimination at 12 months was 9.5 mm (range, 7–12 mm).

Mild joint-space narrowing was observed on the available 12-month radiographs. No conclusion regarding long-term joint viability was drawn from the radiographic findings.

### Secondary procedures and material-related complications

No additional secondary procedures, including flap debulking, scar revision, or tenolysis, were performed after postoperative day 28 during the 12-month follow-up. No clinically evident foreign-body reaction, sterile seroma, delayed wound healing, or surgical-site complication related to the carboxymethylcellulose–hyaluronan adhesion barrier was observed.

### Descriptive context within published series

Selected functional outcomes reported in published thumb-reconstruction series are presented in Table 2 solely to provide descriptive clinical context. Considerable variation was observed across the literature in QuickDASH scores, pinch strength, and IP motion. The published studies differed substantially in injury mechanism, amputation level, reconstructive technique, patient selection, follow-up duration, rehabilitation protocol, and outcome-measurement methodology.

**Table 2:** Selected functional outcomes reported in published thumb reconstruction series for descriptive context.

Study	Pinch Strength (kg)	IP Flexion Arc (°)	QuickDASH Score	Extension Arc (°)
Unglaub et al. (8)	2.5 kg ± 1.05	12 ± 8.4	16.7 ± 15	Included within total motion arc
Chen et al. (9)	3.3 ± 0.8	35.6 ± 5.4	21.0 ± 6.9	Not separately reported
Ciclamini et al. (10)	1.6 ± 0.4	38.2 ± 6.0	14.3 ± 3.6	Not separately reported
David et al. (11)	2.1 ± 0.45	32.5 ± 7.0	20.3 ± 5.6	Not separately reported
Dickey et al. (12)	2.5 ± 0.8	33.0 ± 4.0	22.5 ± 6.0	Not separately reported
Giardi et al. (13)	3.3 ± 1.1	38.7 ± 6.2	15.2 ± 3.9	Not separately reported
Haas et al. (14)	2.9 ± 0.6	50 ± 9	11.3 ± 6.9	Not separately reported
Jia et al. (15)	4.9 ± 0.6	30 ± 11.5	4 ± 1.0	5 ± 2.5 (0–10)
Lin et al. (16)	4.05 ± 0.35	33 ± 5.6	15.4 ± 8.6	Not separately reported
Losco et al. (17)	2.9 ± 0.6	41.6 ± 10.3	11.3 ± 6.9	Not separately reported
Mahmut et al. (18)	2.1 ± 0.7	41.6 ± 6.3	16.0 ± 2.1	Not separately reported
Ma et al. (19)	4.2 ± 0.4	33.2 ± 2.1	12.7 ± 3.4	Not separately reported
Cam et al. (20)	4.65 ± 0.57	65 ± 4.6	2.55 ± 2.5	3.75 ± 4.8
Ince et al. (21)	3.23 ± 0.31	52 ± 7.9	5.1 ± 6.4	Not separately reported

IP, interphalangeal; QuickDASH, Disabilities of the Arm, Shoulder and Hand (quick version). Values are mean ± SD.

Published studies differed substantially in injury mechanism, amputation level, reconstructive technique, patient selection, follow-up duration, rehabilitation protocol, and outcome-measurement methodology. The reported values are presented solely for descriptive clinical context and were not pooled or statistically compared with the present cohort.

Accordingly, the outcomes of the present four-patient cohort are shown alongside selected published series for descriptive context only. The published studies were not treated as a control group, their results were not pooled, and no formal statistical comparisons were performed. The observed numerical differences should therefore not be interpreted as evidence of superiority, equivalence, or comparative effectiveness.

## Discussion

Finger amputation remains a frequent and clinically significant consequence of hand trauma, and when technically feasible, microsurgical replantation continues to represent the preferred reconstructive strategy. In situations where thumb replantation fails intraoperatively, heterotopic groin-pocket salvage may provide a biologically rational salvage approach by preserving viability of the amputated segment within a stable, well-vascularized environment that permits delayed staged tendon and skeletal reconstruction. This approach may be considered in selected high-energy crush or avulsion injuries when viable distal tissue remains available but endothelial disruption and poor vessel quality limit the likelihood of successful repeated microsurgical revision. Under such conditions, temporary heterotopic implantation may prevent immediate loss of the digit while creating an opportunity for delayed reconstruction under more favorable biologic conditions.

Contemporary series have reported digital replantation survival rates approaching 90%, with functional recovery generally ranging between 50% and 90% (22–24). Because the thumb contributes approximately 40%–50% of overall hand function, preservation or restoration of thumb function remains a central reconstructive priority (19). Complex amputations associated with extensive soft-tissue destruction tend to demonstrate lower survival rates and require more resource-intensive postoperative management. Nevertheless, advances in microsurgery and evolving patient expectations have expanded indications for salvage through approaches including short-segment replantation, replantation combined with flap coverage, thumb reconstruction (25), and, in selected cases, cryopreservation followed by delayed replantation (26).

Godina and colleagues first described heterotopic implantation as a temporizing limb-salvage strategy in 1986 (6). Subsequent reports have supported the role of heterotopic replantation in selected extremity and digital injuries, particularly in cases involving severe soft-tissue loss, uncertain tissue demarcation requiring urgent revascularization, polytrauma with hemodynamic instability precluding prolonged microsurgery, or heavily contaminated wounds associated with major tissue destruction (27–29). In severe digital trauma, temporary heterotopic implantation may improve tissue preservation and facilitate staged reconstruction (19).

In the present four-patient series, measurable thumb motion, pinch strength, and sensibility were observed at 12 months, together with a mean QuickDASH score of 3.75. Functional outcomes were assessed using the QuickDASH, a validated 11-item instrument evaluating upper-extremity disability and symptoms (30), whereas aesthetic satisfaction was evaluated using a study-specific Likert scale (31). These findings describe the postoperative status of this highly selected cohort but should not be interpreted as evidence of comparative effectiveness or generalizable clinical performance.

### Principal findings and clinical relevance

At one year, the cohort had a mean QuickDASH score of 3.75, mean tip-to-tip pinch strength of 4.375 kg, corresponding to 96.25% of the contralateral side, and mean IP flexion of 71.25°. Full MCP extension was present in all patients, and two patients demonstrated mild IP hyperextension. These measurements indicate that functional thumb use was retained at the available follow-up; however, the small cohort, lack of an internal comparator, and absence of a fully external centralized assessment preclude broader conclusions regarding effectiveness or reproducibility. The measurements observed in the present cohort can be considered within the broad spectrum of outcomes reported in selected thumb-reconstruction series. However, differences in injury mechanism, amputation level, reconstructive technique, patient selection, rehabilitation, follow-up duration, and outcome-measurement methodology prevent valid direct comparison. The published values are therefore presented solely as descriptive clinical reference points and do not establish superiority, equivalence, or comparative effectiveness of the present technique.

The observed recovery pattern is biomechanically consistent with the reconstructive objectives of the technique. Preservation of the distal phalanx segment maintains both a skeletal scaffold and the native tendon insertion sites, while staged FPL and EPL reconstruction facilitates restoration of a stable pinch post together with a functional motion arc. In this context, an IP flexion arc in the 70° range provides a practical envelope for object manipulation, whereas preserved pinch strength supports activities requiring opposition and fine prehension. The functional observations in this series are consistent with the intended biomechanical rationale of preserving the distal phalanx and native tendon insertion sites. Nevertheless, the present study cannot determine which component of the staged reconstruction contributed most to the observed outcomes.

A carboxymethylcellulose–hyaluronan adhesion barrier (SEPRAFILM®) was used off-label around the peri-IP region during staged reconstruction. Its use was informed by experimental evidence suggesting reduced adhesion formation and fibrosis in an animal arthrofibrosis model, together with broader principles concerning fibrocontractive wound healing (32, 33). However, all four patients received the barrier, and no untreated control group was available. Consequently, the preserved IP motion cannot be attributed to SEPRAFILM®, and its clinical efficacy in this reconstructive setting remains undetermined. No foreign-body reaction, sterile seroma, delayed wound healing, or surgical-site complication attributable to the material was observed during the available follow-up. Formal digital nerve coaptation was not performed because preservation of vascularized tissue and construct stability were prioritized during the staged salvage procedure. At 12 months, mean static two-point discrimination was 9.5 mm. In the absence of formal nerve repair, the observed sensibility may reflect collateral reinnervation from adjacent innervated tissues, spontaneous axonal ingrowth across the reconstructed soft-tissue interface, and central sensory adaptation. The present study was not designed to determine the mechanism of reinnervation, and these findings should be interpreted as evidence of useful protective sensibility rather than restoration of normal digital nerve function. Although no formal sensory re-education protocol was implemented, repeated use of the reconstructed thumb during daily activities may have contributed to functional sensory adaptation.

Mild joint-space narrowing was observed on the available 12-month radiographs. Although no gross articular collapse or advanced degenerative change was evident at this time point, 12 months is insufficient to establish long-term joint viability. Progressive joint-space loss, secondary osteoarthritis, arthrofibrosis, osteonecrosis or resorption of the preserved phalangeal segment, tendon adhesion, instability, and functional decline may become apparent only after several years. Continued clinical and radiographic follow-up is therefore required.

### Context within the literature

The literature on thumb reconstruction after traumatic loss encompasses a broad spectrum of reconstructive strategies, including replantation, toe-to-thumb transfer, index pollicization, and composite flap-based reconstruction. Direct comparison across studies is limited by substantial differences in injury mechanism, amputation level, timing of reconstruction, surgical technique, rehabilitation protocol, follow-up duration, and functional outcome assessment.

Published thumb-reconstruction series report considerable variation in patient-reported disability. QuickDASH scores in several studies have generally ranged between approximately 11 and 22 points (9, 12), whereas selected cohorts have reported lower values, including approximately 4 ± 1 in the series by Jia et al. (15) and 2.6 ± 2.5 in the series by Cam et al. (20). The mean QuickDASH score in the present cohort was 3.75. However, these numerical values are presented solely for descriptive context and should not be interpreted as evidence of superior or equivalent patient-reported outcomes because of substantial differences between the respective patient populations and reconstructive procedures.

Reported pinch strength also varies considerably across published series, generally ranging from approximately 1.6–4.9 kg (15, 20). The mean tip-to-tip pinch strength in the present cohort was 4.38 kg, corresponding to approximately 96% of the contralateral side. Similarly, reported postoperative IP flexion varies across the literature, with several studies reporting values between approximately 30° and 52° (14, 21). The mean IP flexion observed in the present cohort was 71.3°. These findings describe the postoperative function observed in the present four patients but cannot be validly compared with published cohorts because of differences in injury pattern, amputation level, joint preservation, reconstruction, rehabilitation, and measurement methodology.

**Figure 4:**
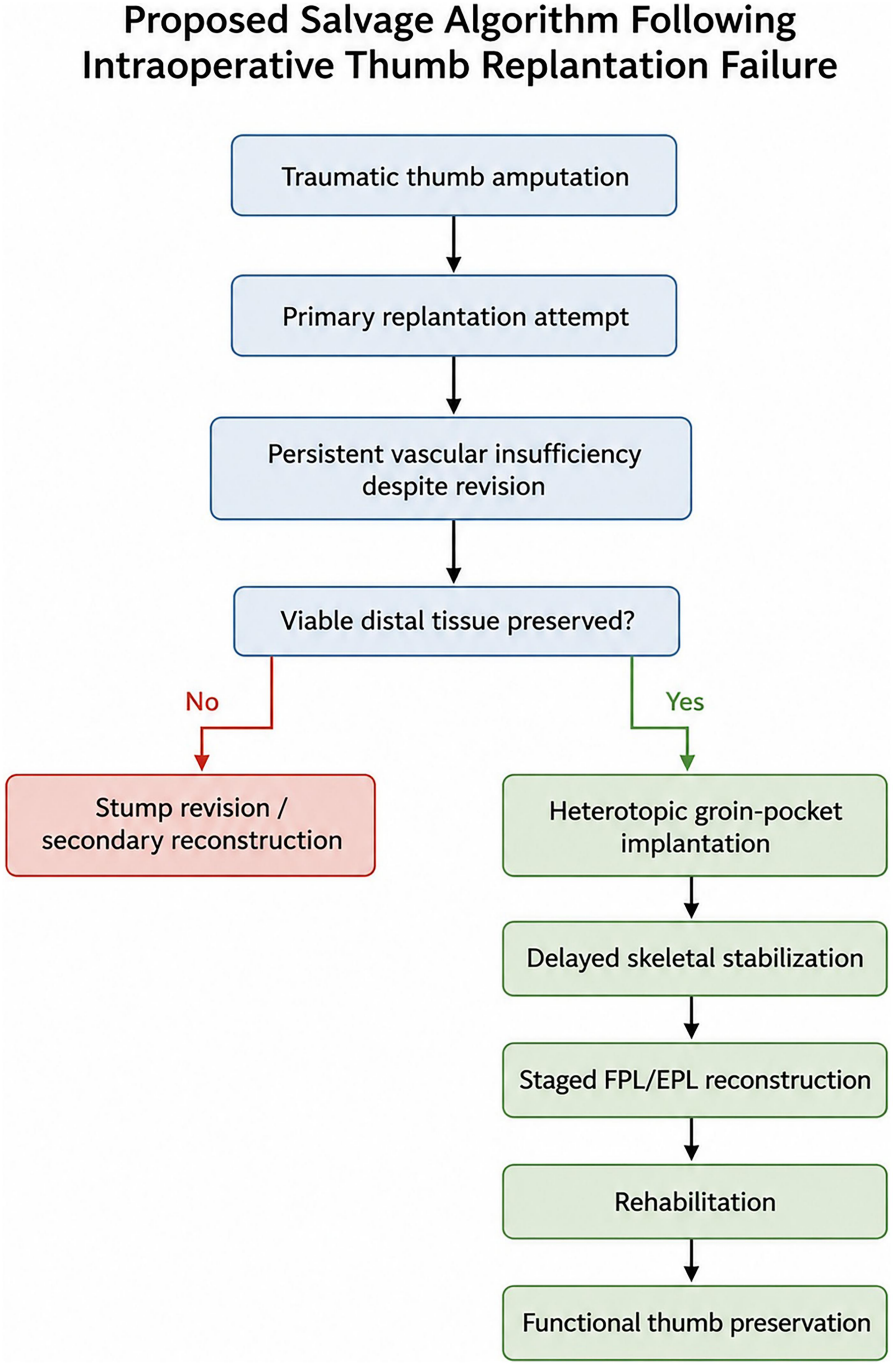
Proposed staged salvage algorithm following intraoperative thumb replantation failure. When persistent vascular insufficiency remains unresolved despite repeated microsurgical revision, assessment of distal tissue viability may guide reconstructive decision-making. In selected patients with preserved distal tissue architecture, staged heterotopic groin-pocket implantation may permit delayed skeletal stabilization, tendon reconstruction, rehabilitation, and subsequent functional thumb preservation. Patients without salvageable distal tissue may require stump revision or alternative secondary reconstructive procedures.

Lo and Wei (34) compared toe-to-hand transfers with digit replantations using the Michigan Hand Outcomes Questionnaire and reported higher composite scores in the toe-transfer cohort. However, the Michigan Hand Outcomes Questionnaire incorporates multiple patient-reported domains and is not directly interchangeable with the objective motion, pinch-strength, sensibility, and QuickDASH measurements used in the present study. Toe transfer and staged heterotopic salvage also represent fundamentally different reconstructive strategies. Preservation of the original distal thumb tissue avoids creation of a new toe-donor site by definition, but the present series does not establish an overall functional or patient-reported advantage over toe transfer or other reconstructive options. Based on the technical sequence and patient-selection principles used in this series, a proposed staged decision-making algorithm for intraoperative thumb replantation failure is illustrated in Figure 4.

### Technical rationale in relation to other salvage strategies

Several established reconstructive strategies exist following failed thumb replantation, including revision amputation, prosthetic adaptation, toe-to-thumb transfer, index pollicization, and secondary flap-based reconstruction (2, 15, 16). However, each approach carries inherent limitations related to donor-site morbidity, altered biomechanics, limited sensibility, or loss of native thumb architecture (2, 34).

In contrast, the present staged heterotopic salvage strategy attempts to preserve the patient's original distal thumb tissue, including the native distal phalanx and preserved FPL/EPL tendon insertion sites. This biologic preservation strategy may facilitate restoration of a functional pinch post while minimizing additional donor morbidity associated with toe transfer procedures (15, 16, 34).

Unlike conventional ectopic implantation reports that primarily emphasize tissue survival, the present series specifically focused on delayed functional reconstruction and objective postoperative functional outcomes, including pinch strength, motion arc, and patient-reported disability (4, 5, 27, 35). This preservation-based approach is technically distinct from reconstructive procedures that replace the missing thumb using tissue from another donor site. However, whether this distinction translates into superior functional or patient-reported outcomes cannot be determined from the present series.

### Mechanistic and technical considerations

Several biologic and technical factors may have contributed to the postoperative function observed in this series. First, temporary ectopic implantation preserves viability of the amputated segment when immediate *in situ* revascularization is either technically unachievable or hemodynamically unreliable. By transferring the amputated part into a stable vascularized environment, definitive skeletal and tendon reconstruction can be deferred until tissue perfusion and soft-tissue conditions become more favorable. This staged strategy may provide additional time for serial debridement, reduction of contamination burden, and planning of secondary reconstructive procedures under optimized biologic conditions.

Second, the groin region provides a pliable and adaptable soft-tissue envelope that can be tailored to approximate contralateral thumb dimensions, thereby facilitating resurfacing and subsequent mobilization. The postoperative rehabilitation protocol may have contributed to tendon excursion, joint motion, and functional adaptation; however, the absence of a comparator group prevents separation of the effects of rehabilitation from those of the surgical reconstruction itself. These principles are consistent with the biologic rationale underlying previous ectopic salvage strategies in complex extremity trauma.

Digital nerve coaptation was intentionally not prioritized during the initial salvage stages because preservation of vascularized viable tissue and restoration of a stable functional thumb post represented the primary reconstructive objectives in the setting of progressive intraoperative vascular insufficiency. Additional microsurgical manipulation required for nerve repair was considered potentially detrimental to flap perfusion and construct stability during the critical early integration phase. Nevertheless, all patients achieved protective sensibility, with a mean static two-point discrimination of 9.5 mm at final follow-up.

Similarly, extensor pollicis longus (EPL) reconstruction was intentionally delayed until late-stage integration of the osteocutaneous construct. This staged approach was designed to minimize dorsal tension across the reconstructed thumb and reduce the risk of vascular compromise during the critical neovascularization period. Delayed tendon reconstruction also facilitated improved soft-tissue stabilization and more controlled postoperative mobilization.

Despite the absence of formal collateral ligament or volar plate reconstruction, no clinically evident lateral or rotational instability was observed at the 12-month examination, and none of the patients reported pain during maximal-effort tip-to-tip pinch testing. Temporary Kirschner-wire fixation, approximation of the residual periarticular tissues, staged tendon balancing, and support from the surrounding groin-flap tissues may have contributed to the observed clinical stability. Nevertheless, stability was assessed manually without instrumented stress testing, and these findings do not establish long-term biomechanical durability.

### Strengths and interpretive cautions

The principal strength of this study is the detailed presentation of a staged salvage technique together with multidimensional 12-month outcome assessment in a rare reconstructive scenario. The literature review was used solely to provide descriptive clinical context rather than a formal comparator. Because published series differed substantially in injury pattern, amputation level, reconstructive strategy, follow-up duration, rehabilitation, and outcome reporting, no pooled estimates or inferential comparisons were generated.

Interpretation of the present findings should nevertheless acknowledge several important sources of variability across the available literature. Published series differ considerably with respect to injury mechanism, severity of soft-tissue destruction, timing of reconstruction, and reconstructive strategy, including replantation, toe transfer, and composite flap-based techniques. In addition, variability in rehabilitation protocols, measurement methodology, and outcome-reporting standards may substantially influence reported postoperative function. Parameters such as goniometric technique, dynamometer calibration, and inclusion or exclusion of extension arcs are inconsistently reported across studies, thereby limiting strict interstudy comparability. Accordingly, the individual published values summarized in Table 2 should be interpreted solely as descriptive clinical reference points rather than performance benchmarks.

### Clinical implications

Persistent intraoperative circulatory failure during thumb replantation may leave the surgeon with limited options after repeated microsurgical revision attempts have failed. In carefully selected patients with preserved viable distal tissue architecture, acceptable wound conditions, and no irreversible proximal tissue destruction, staged heterotopic groin-pocket salvage may represent a technically feasible option rather than immediate revision amputation. The present series demonstrates the feasibility of this approach and describes functional use at 12 months; however, it does not establish comparative effectiveness, superiority over other reconstructive strategies, or criteria for broader clinical application.

As emphasized in the contemporary trauma literature, restoration of durable function after severe thumb injury remains challenging, particularly in crush-avulsion patterns associated with compromised vascular integrity (35). The motion, pinch strength, sensibility, and patient-reported outcomes observed in these four patients support further investigation of the technique but should not be interpreted as justification for routine adoption. Patient selection should remain cautious, and patients should be counselled regarding the staged nature of the reconstruction, the need for prolonged rehabilitation, and the uncertainty surrounding long-term joint and skeletal viability.

Because intraoperative thumb replantation failure is uncommon, larger single-center cohorts may be difficult to obtain. Multicenter collaboration, independent outcome assessment, and longer clinical and radiographic follow-up will therefore be important to define the reproducibility, indications, complications, and long-term durability of this salvage strategy.

### Limitations

This study has several important limitations. First, the cohort consisted of only four highly selected patients, all with amputations at the IP joint level. This anatomical homogeneity and small sample size substantially limit generalizability to more proximal injuries, contaminated wounds, or cases with more extensive soft-tissue destruction. The study had no internal control group, and the selected published series were used solely to provide descriptive clinical context. Heterogeneity in injury pattern, reconstructive technique, follow-up duration, rehabilitation, and outcome assessment precluded formal pooling or direct comparative interpretation.

Objective outcome assessments were performed independently by the operating senior microsurgeon and by a second hand surgeon who had not participated in the patients' treatment and was blinded to the operating surgeon's measurements and to the patients' previously recorded functional outcome data. This approach reduced the risk of observer and measurement bias. Nevertheless, the assessments were not performed by a fully external centralized evaluation team, and complete blinding to the visible postoperative reconstruction was not possible. Joint stability was assessed clinically rather than by instrumented stress testing, and pain during pinch was recorded without a validated pain scale.

Follow-up was limited to 12 months, which is insufficient to establish the durability of a staged joint-preserving reconstruction. Progressive joint-space loss, secondary osteoarthritis, arthrofibrosis, osteonecrosis or resorption of the preserved phalangeal segment, tendon adhesion, late instability, and functional decline may become apparent only after several years. Mild joint-space narrowing was already present at the available follow-up and should not be interpreted as evidence of preserved long-term joint viability.

All patients received the carboxymethylcellulose–hyaluronan adhesion barrier, and no untreated control group was available; therefore, the observed IP motion cannot be attributed to this adjunct. In addition, the mechanism of sensory recovery could not be determined in the absence of formal nerve coaptation or electrophysiological assessment. Aesthetic satisfaction was evaluated using a study-specific Likert scale, and rehabilitation may have materially influenced the reported functional outcomes. Larger multicenter studies with independent outcome assessment and longer clinical and radiographic follow-up are required.

## Conclusions

This four-patient retrospective technical case series demonstrates the feasibility of staged heterotopic groin-pocket salvage after intraoperative failure of thumb replantation at the IP joint level. At 12 months, the patients retained measurable motion, pinch strength, protective sensibility, and clinical stability without pain during maximal-effort pinch. However, the small and anatomically homogeneous cohort, absence of an internal comparator, incomplete blinding to the visible reconstruction, and limited follow-up preclude conclusions regarding comparative effectiveness, reproducibility, or long-term joint viability. The technique should therefore be regarded as a preliminary salvage option for highly selected cases and requires validation through larger multicenter experience and longer clinical and radiographic follow-up.

## Data Availability

The original contributions presented in the study are included in the article/Supplementary Material, further inquiries can be directed to the corresponding author.

## Supplementary material

Supplementary Figure 1 Additional clinical views from the staged heterotopic groin-pocket salvage procedure. (a) Preoperative design of the ipsilateral groin pocket based on contralateral thumb dimensions. (b) Primary closure of the proximal thumb stump following heterotopic implantation. (c) Volar view of the reconstructed thumb at 12 months demonstrating stable soft-tissue coverage and mild residual shortening.
